# Evaluation of the Antimicrobial Potential of Malaysian Kelulut Honeydew Honey Against Acne Pathogens

**DOI:** 10.1007/s00284-026-04866-6

**Published:** 2026-04-10

**Authors:** Siau Wui Chin, Adzzie-Shazleen Azman, Michelle Khai Kun Yap, Ji Wei Tan

**Affiliations:** School of Science, Monash University Malaysia, Jalan Lagoon Selatan, Bandar Sunway, Subang Jaya, 47500 Selangor Malaysia

## Abstract

Acne is the eighth most prevalent chronic skin disease globally. The increased emergence of antibiotic resistance and chemical-induced side effects make acne treatments challenging. Hence, a natural remedy, Malaysian Kelulut honeydew honey, was proposed in this research due to its beneficial properties and the absence of antibiotic resistance. An in vitro efficacy of honey was evaluated against acne-associated microbes (*Cutibacterium acnes*, *Staphylococcus aureus*, *Staphylococcus epidermidis*, methicillin-resistant *Staphylococcus aureus* and *Candida albicans*) via broth microdilution assay. The honey exhibited inhibitory and bactericidal effects against *Cutibacterium acnes* at 20% and 40%, respectively, while only inhibitory effects were observed against other microbes. *Cutibacterium acnes*, the most susceptible bacteria in this study, was subjected to time-kill assays to assess the time- and concentration-dependent effects of honey. The honey’s inhibitory (20% honey) and killing (40% honey) kinetics started at the 12th and 4th hour, respectively. Morphological alterations in *Cutibacterium acnes* following treatments were observed via field emission scanning electron microscopy (FESEM), revealing the deformation of the rod-shaped *Cutibacterium acnes* and filamentation occurrence. Through liquid chromatography mass spectrometry (LCMS), the potential phytochemicals responsible for honey’s antimicrobial activity were identified to be purine, pteridine, theobromine, nebularine, and nigerose (sakebiose). To conclude, Malaysian Kelulut honeydew honey is a promising anti-acne agent by targeting *Cutibacterium acnes*. To date, the antimicrobial activities of Malaysian Kelulut honeydew honey have been underexplored, especially against *Cutibacterium acnes*. Notably, this is the first report of honey inducing filamentation in any *Cutibacterium acnes* strain, suggesting a possible mechanism of action of honey.

## Introduction

Acne is the eighth-most prevalent skin disease involving inflammation in the pilosebaceous unit, with an estimated 9.38% prevalence rate globally [1, 2]. In 2016, approximately 680 million people worldwide were reported to have acne, marking a 10% increase from 612 million people in 2006 [3]. Acne is generally manifested as inflammatory or non-inflammatory skin lesions, which include open (blackheads) and closed comedones (whiteheads) and papules, pustules and more severe nodules and cysts, respectively [4]. Besides being influenced physically, acne can affect the mental health, social life and quality of life of an individual due to embarrassment, increased risk of depression, anxiety and suicide attempts [5, 6].

The four main pathophysiologies of acne include hyperserborrhea, hyperkeratinization, colonization of pathogenic *Cutibacterium acnes* (*C. acnes*) and inflammation [7]. Hyperserborrhea (sebum oversecretion) is the primary factor in acne development, which, together with hyperkeratinization, forms a keratotic plug that obstructs the pilosebaceous ducts, leading to comedone development [8]. Persistent sebum accumulation and the resulting hypoxic environment of the folliculopilosebaceous unit favour the colonization of anaerobic *C. acnes*. Pathogenic *C. acnes* is the secondary factor of acne, as it induces extracellular matrix (ECM) degradation and releases inflammatory cytokines, potentiating inflammatory skin lesions [9]. Skin barrier perturbation shifts commensal skin microbes such as C. acnes (dominant cutaneous bacteria), *Staphylococcus epidermidis (S. epidermidis)*, and the fungi *Malassezia* spp. into opportunistic pathogens, leading to skin microbiota dysbiosis and increased skin infections by other microbes such as *Staphylococcus* spp. and *Candida* spp.

Antibiotics such as clindamycin, erythromycin, chloramphenicol and tetracycline were initially effective against pathogenic *C. acnes* to control inflammatory acne until the increased emergence of antibiotic resistance that has become a global health threat by causing approximately 1.27 million deaths worldwide [9, 10]. Increased resistance of *C. acnes* to erythromycin, clindamycin and tetracycline has been reported with resistance rates of 15%, 4% and 2%, respectively [11]. The increased emergence of antibiotic resistance was also present in other bacteria (*S. aureus*, *S. epidermidis* and MRSA) [12, 13]. Besides, topical chemical ointments such as salicylic acid and benzoyl peroxide are frequently accompanied by side effects such as skin dryness, local burning and skin irritation [9]. Hence, natural remedies were revisited to reduce antibiotic reliance and mitigate the adverse effects of chemical acne treatments.

Historically, honey first acted as a food source and was used in the medicinal field, specifically in wound healing before 2000 BCE [14]. Remarkably, honey has not been documented to develop microbial resistance and exhibits a broad spectrum of antimicrobial activities [14, 15]. Recently, researchers have redirected their interest from the extensively studied Manuka honey (sting bee honey) to the less-explored stingless bee honey due to the comparable or greater antimicrobial activity [16]. This could be attributed to propolis in the cerumen pots of stingless bee nests, which greatly infuses the stingless bee honey with plant-derived antimicrobial compounds [17]. Furthermore, stingless bee honey was reported to have a broader spectrum of inhibitory activities against Gram-positive, Gram-negative and antibiotic-resistant bacterial strains [15]. Despite extensive research on the antimicrobial properties of various honeys, the effect of Malaysian Kelulut honey on acne-causing bacteria, especially *C. acnes*, remains unexplored.

Therefore, our research highlighted the antimicrobial potential of stingless bee honey, locally known as Kelulut honeydew honey, against acne-associated microbes. We evaluated its efficacy and identified the most susceptible microorganism for further analysis. FESEM was employed to gain preliminary insights into the morphological alterations induced by the honey on the selected microbe. LCMS analysis was carried out to identify the potential phytochemicals in honey responsible for its antimicrobial activity. This study aims to explore a natural alternative to acne treatments that reduces antibiotic resistance and the adverse side effects of chemicals.

## Materials and Methods

### Materials

The media Mueller-Hinton agar (MHA), Mueller-Hinton broth (MHB), brain heart infusion agar (BHIA), brain heart infusion broth (BHIB), malt extract agar (MEA) and malt extract broth (MEB) were purchased from Himedia (India). The antibiotics chloramphenicol (CHL) and meropenem (MEM) were purchased from Nacalai Tesque (Japan) and Merck (Germany), respectively. The antifungal cycloheximide (CHX) was purchased from VWR Life Science (USA). Phosphate buffered saline (PBS) tablets were purchased from GoldBio (USA).

### Microbe Cultivation and Inoculation

The tested microbes were Gram-positive bacteria *Staphylococcus aureus* (*S. aureus*) (ATCC 25923), methicillin-resistant *Staphylococcus aureus* (MRSA) (DSM 11729), *Staphylococcus epidermidis* (*S. epidermidis*) (ATCC 12228) *Cutibacterium acnes* (*C. acnes*) (DSM 16379) and Gram-positive fungus *Candida albicans* (*C. albicans*) (ATCC 10231). *S. aureus*, MRSA and *S. epidermidis* were subcultured on MHA and incubated for 18–24 h at 37 °C. *C. acnes* was subcultured on BHIA and incubated for 72 h at 37 °C. *C. albicans* was subcultured on MEA and incubated for 48 h at 30 °C unless stated otherwise. A loopful of microbial cells was taken from the agar culture and transferred into the respective broth to prepare the microbial inoculum. Unless stated otherwise, MHB was used for bacteria *S. aureus*, *S. epidermidis* and MRSA; BHIB for bacteria *C. acne*s and MEB for fungus *C. albicans* for microbial inoculation. The microbial broth cultures were then incubated in a shaking incubator at 37 °C for the bacteria and 30 °C for the fungus.

### Honey Origin and Preparation

Stingless bee honey was obtained from a collaborator (Eco Bee Shop Sdn. Bhd.) from Gemas, Johor. The honey was harvested by the bee species *Heterotrigona itama* (*H. itama*), with the nectar source being the honeydew of the monofloral *Acacia mangium* tree. A stock concentration of 80% (v/v) of the raw honey was prepared based on a 4:5 ratio (volume of raw honey: total volume of raw honey and sterile water, respectively). The unfiltered 80% (v/v) honey was mixed with a vortex mixer and filtered with a 0.22 μm sterile nylon syringe filter (GVS filter technology, USA) and 10 cc/mL syringe (Terumo, Philippines). The filtered honey was stored at 4 °C until further use. The pH of the filtered 80% honey stock solution was measured using a benchtop Laqua pH meter (Horiba, Japan).

### Minimum Inhibitory Concentration (MIC) Analysis

The bacterial culture was prepared based on the McFarland standard and further diluted to achieve 1 $\:\times\:$10^5^ CFU/mL for the microdilution method, according to CLSI guideline. However, the inoculum size for *C. acnes* was optimised to 1 × 10^8^ CFU/mL due to its slow-growing nature, in order to ensure sufficient bacterial growth and visible turbidity after the 3-day incubation period. The working concentrations of honey were 40%, 20%, 10%, 5%, and 2.5% (v/v). Controls (negative, positive, sterile and colour controls) were included. The 96-well plate was incubated at 37 °C for 24 h (*S. aureus*, *S. epidermidis* and MRSA), 72 h (*C. acnes*), and at 30 °C for 48 h (*C. albicans*). This assay was performed in triplicate. MIC was visually determined as the first clear well observed at the lowest concentration.

### Minimum Bactericidal/Fungicidal Concentration Analysis

The spread plate method was employed and performed in duplicates. A volume of 0.1 mL of all the negative wells (no microbial growth), negative control (NC) and the first positive well (with microbial growth) were drawn and transferred onto the respective agar plates. A sterile glass spreader (rinsed with 70% ethanol, flamed to dry and allowed to cool down) was used to immediately spread the suspension evenly over the agar surface. The agar plates were incubated at 37 °C for 24 h (*S. aureus*, *S. epidermidis* and MRSA), 72 h (*C. acnes*), and at 30 °C for 48 h (*C. albicans*). The absence of growth on the plate indicated MBC/MFC of the treatment.

### Time-Kill Assay

*C. acnes* was subjected to a further time-killing assay and conducted in duplicates. Microbial inoculum was prepared freshly for microbial growth in the early logarithmic phase. *C. acnes* was tested against 1 $\:\times\:\:$MIC and 1 $\:\times\:$ MBC of honey compared to MEM, the positive control (PC) (at MIC = 1.25 µg/mL and MBC = 5 µg/mL) and NC. The honey-treated samples, positive and negative controls, were incubated at 37 °C. At the time points 0, 2, 4, 6, 12 and 24 h, ten-fold dilution was done, and 100 µL from the desired dilutions was drawn for spread plating. All plates were then incubated for 5 days at 37 °C. After the incubation period, plates with 30 to 300 colonies were counted. The log_10_ (CFU/mL) was calculated based on the formula below, and a graph of log_10_ (CFU/mL) was plotted against time (hours).

$$\rm \log_{10}(CFU/mL) = Average\ of\ \log_{10} [CFU/(dilution\ factor \times volume\ of\ aliquot)]$$

### Field Emission Scanning Electron Microscopy (FESEM)

*C. acnes* bacterial suspension was adjusted to meet 0.5 McFarland standard (~ 1$\:\times\:$10^8^ CFU/mL) and centrifuged at 5000 rcf for 15 min to obtain the pellet. The bacterial pellets were treated with PC at MIC (1.25 µg/mL MEM) and MBC (5 µg/mL MEM) and honey at MIC (20% (v/v)) and MBC (40% (v/v)). The untreated bacterial pellets (NC) were added with 1$\:\times\:$ phosphate buffered saline (PBS). The samples were incubated for 24 h at 37 °C. After incubation, the samples were centrifuged at 5000 rcf for 15 min to obtain the pellet. The pellets were washed with 1x PBS twice and fixed with 2.5% glutaraldehyde (GA) at 4 °C overnight. The fixed samples were washed with 1x PBS twice before being dehydrated gradually with 30%, 50%, 70%, 90%, 100%, 100% and 100% ethanol in sequence for 10 min in each solution. The samples of 15 µL were then aliquoted out onto a coverslip and dried in a desiccator overnight. The samples were platinum-coated with the sputter current and time of 30 mA and 35 s, respectively. Observation was done under a field emission scanning electron microscope (FESEM) (Hitachi SU8010, Japan).

### Liquid-Chromatography Mass Spectrometry (LCMS)

The LCMS analysis of the honey was carried out using the Agilent 1290 Infinity LC system coupled to Agileng 6520 Accurate-Mass Q-TOF mass spectrometer with dual ESI source. The column employed was Agilent Eclipse XDB-C18 Narrow-bore, 150 mm $\:\times\:$ 2.1 mm, 3.5-micron (P/N 930990-902). Temperatures for the column and auto-sampler were kept at 25 and 4 °C, respectively. The flowrate was 0.5 mL/mins. The mobile phases used were 0.1% formic acid in water (solvent A) and 0.1% formic acid in acetonitrile (solvent B), whereas the injection volume was 1 µL. There was a 25 min run and a 5 min recovery period. Electrospray ion source in both the negative and positive mode was employed. Full scan MS analysis was performed over the m/z 100–3200 range. Agilent MassHunter Qualitative Analysis B.07.00 was used to process the data with standard processing parameters. The chemical composition of the honey was identified by comparing their mass spectra with those of the reference spectra in the Metlin database (Metlin_AM_PCDL-N-170502.cdb) using the SM QTOF guidelines, with search parameters: 5 ppm match tolerance ions with positive ions include + H, +Na, +K an +NH4, while negative ions include -H and -Cl. To narrow down the compound lists from the raw data, the compounds present in the blank were removed, and only compounds with a database difference (DB Diff) within the range − 2 to + 2 were considered.

### Statistical Analysis

Experimental results were reported as mean ± standard deviation (sd). One-way analysis of variance (ANOVA) was used to compare the mean differences between all three tested groups (NC, PC and honey treatment). Two-way ANOVA was used to determine the significant effects due to multiple factors (time and treatment groups) and the interaction between the two factors. Tukey post-hoc test followed if ANOVA showed significance to determine the pair of groups with significant mean differences. The GraphPad Prism version 8.0.2 software was used for statistical analyses. Statistical significance was defined as *P* < 0.05.

## Results and Discussion

### MIC and MBC/MFC of Honey

Broth microdilution and MBC/MFC assays demonstrated that Malaysian Kelulut honeydew honey exhibited inhibitory activity against all tested microbes. The positive controls used were as listed: 0.625 µg/mL CHL (against *S. epidermidis*); 1.25 µg/mL CHL (against *S. aureus* and MRSA); 1.25 µg/mL MEM (against *C. acnes*) and 1.25 µg/mL CHX (against *C. albicans*). The pH of the 80% filtered honey measured ranged from 3.34 to 3.65. Honey showed uniform MIC values of 20% (v/v) against all bacterial strains but was bactericidal only against *C. acnes* (MBC = 40% (v/v)). Among the tested microorganisms, *C. albicans* required the highest honey concentration for growth inhibition (MIC = 40% (v/v)) (Table 1).

Our results are inconsistent with the existing research where Australia stingless bee honey exhibited MIC of 4–16% (v/v) against *S. aureus*, MRSA and *S. epidermidis* [17]. *S. aureus* and MRSA demonstrated MIC/MBC of 6.25%/12.5% (v/v) for Tazma stingless bee honey [18]. Notably, *H. itama* honey in a study showed MIC and MBC values ranging from 1.56 to 6.25% (w/v) and 3.125 to 6.25% (w/v), respectively, against *S. aureus* [19]. Omar et al. (2019), who also tested on *H. itama* honey, showed MIC/MBC values of 6.25%/25% (v/v) and 12.5%/25% (v/v) against MRSA and *S. aureus*, respectively [20]. In a study conducted by Boorn et al. (2010), it was observed that *C. albicans* exhibited comparable MIC/MFC values (≥ 32% (v/v)) to our research [17]. This similarity could be attributed to the same fungal strains employed. Honey exerted more potent inhibitory effects against the bacteria than fungi (20% vs. 40%), postulating the nature of fungi with greater acidic tolerance than bacteria. For instance, *C. albicans* can grow from pH 2 to 10 [21].

The disparity in the MIC or MBC values could be due to the variation of stingless bee honey types and microbial strains, the sensitivity of the tested microbes to the honey and differences in experimental methodologies. The variation in the antimicrobial capacity in stingless bee honey of the same bee species (*H. itama*) can be attributed to the different geographical regions, botanical sources and seasonal conditions of honey collection, as well as the honey processing and storage conditions [22].

**Table 1:** MIC (% (v/v)) and MBC/MFC (% (v/v)) values of honey against *S. epidermidis*,* S. aureus*, MRSA, *C. acnes* and *C. albicans*

Microbes	MIC (%)	MBC (%)	MFC (%)
*S. epidermidis*	20	> 40	N/A
*S. aureus*	20	> 40	N/A
MRSA	20	> 40	N/A
*C. acnes*	20	40	N/A
*C. albicans*	40	N/A	> 40

### Time-Kill Assay

**Fig. 1:**
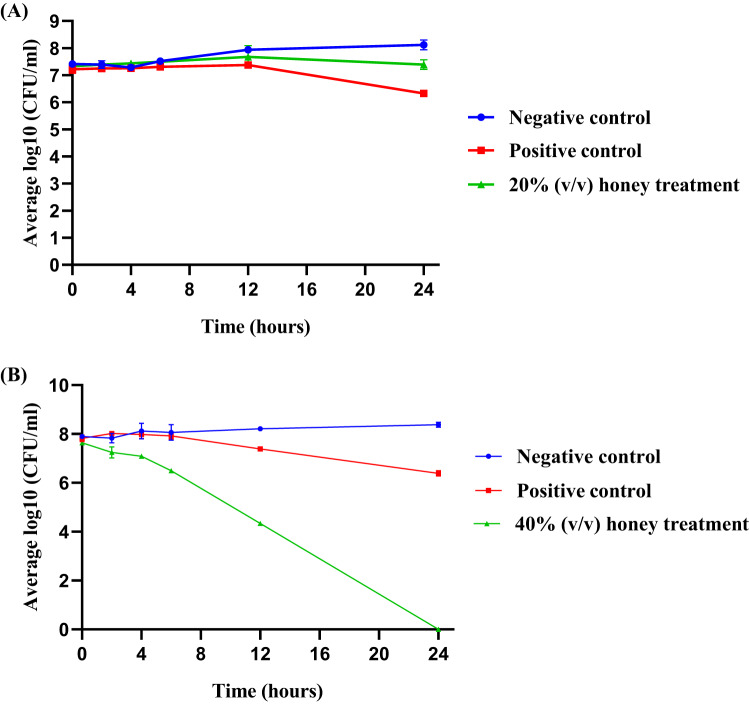
Time-kill curves of *C. acnes* exposed to (**A**) honey at 1 $\:\times\:$ MIC (20%), MEM as PC at 1.25 µg/mL and (**B**) honey at 1 $\:\times\:$ MBC (40%), MEM as PC at 5 µg/mL. NC indicates bacterial growth without any treatment. Results were reported as an average of log10(CFU/mL)) ± standard deviation (error bars). Significant differences between and among groups at *p* < 0.05 were analyzed using Two-way ANOVA. *MIC*,minimum inhibitory concentration*; MBC*,minimum bactericidal concentration*; MEM*,meropenem*; PC*,positive control*; NC*,negative control

Given its highest susceptibility towards honey, *C. acnes* was selected for further evaluation using time-kill kinetics. There was no significant difference in mean log_10_(CFU/mL) for 20% (v/v) honey treatment compared to PC and NC (*p* > 0.05) (Fig. 1(A)). On the contrary, Fig. 1(B) showed a significant decrease in CFU/mL for honey treatment (40% (v/v)) (*p* < 0.05).

Bactericidal and bacteriostatic activities are defined as the reduction of ≥ 3 log_10_ (CFU/mL) and < 3 log_10_ (CFU/mL), respectively, compared to the original bacterial inoculum [23]. The inhibitory effect of 20% honey was evident from the 12th hour, persisting up to 24 h. In contrast, treatment with 40% (v/v) honey produced rapid and sustained bactericidal activity, with a reduction exceeding 3 log₁₀(CFU/mL) observed as early as the 4th hour and an approximate 8 log₁₀(CFU/mL) reduction after 24 h (Fig. 1(B)).

These results demonstrate concentration- and time-dependent antimicrobial effect of Kelulut honey against *C. acnes* and corroborate the MIC and MBC findings. The pronounced bactericidal activity at higher concentrations highlights the potential of this honey to effectively inhibit *C. acnes* overcolonization, a key contributor to acne pathogenesis.

### Field Emission Scanning Electron Microscopy (FESEM)

**Fig. 2:**
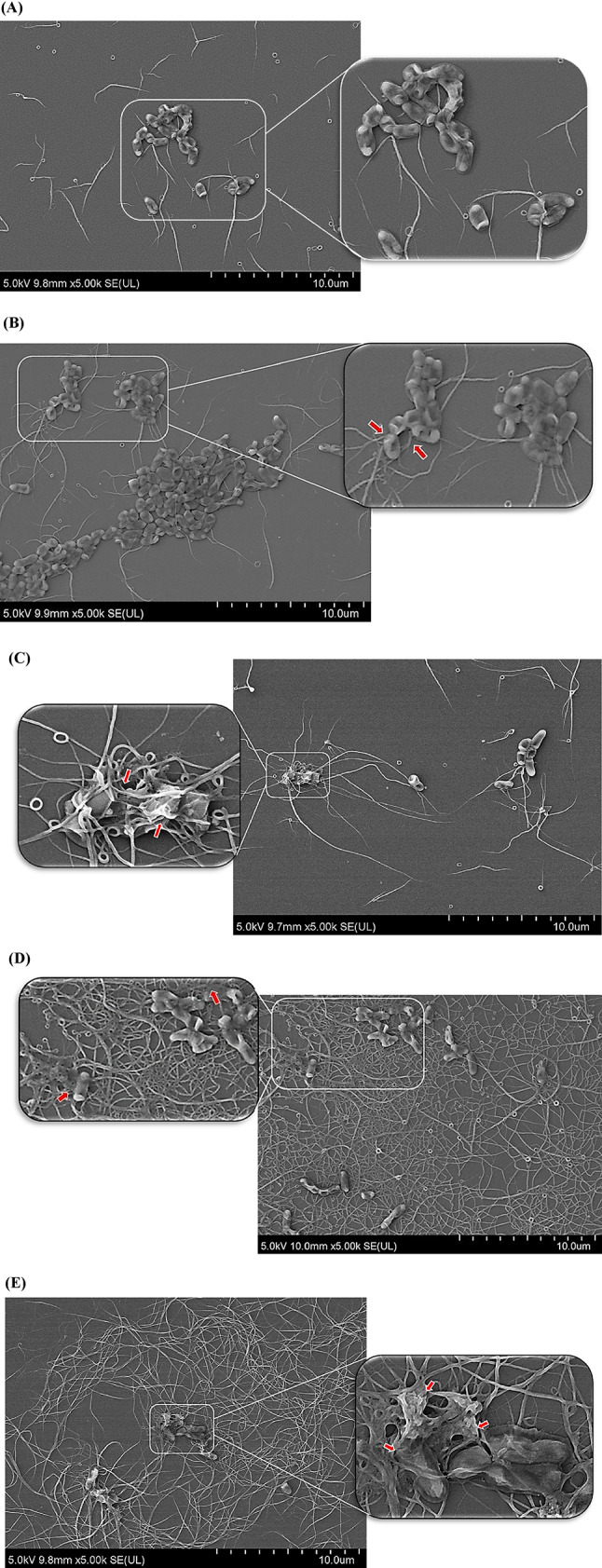
FESEM images of the antimicrobial effect of stingless bee honey against *C. acnes* under the magnification power of 5000x: (**A**) *C. acnes* NC; (**B**) *C. acnes* treated with PC of MEM at MIC of 1.25 µg/mL; (**C**) *C. acnes* treated with 20% (v/v) honey (MIC); (**D**) *C. acnes* treated with PC of MEM at MBC of 5 µg/mL; (E) *C. acnes* treated with 40% (v/v) honey (MBC). Red arrows indicated the shrinkage and deformation of *C. acnes* bacterial cells, which were then extended to form filaments (long, threadlike structures). *MIC*,minimum inhibitory concentration*; MBC*,minimum bactericidal concentration*; MEM*,meropenem*; PC*,positive control*; NC*,negative control

FESEM analysis revealed marked morphological alterations in *C. acnes* cells following honey exposure. Untreated cells exhibited intact, short rod-shaped morphology, whereas treated cells displayed cell shrinkage, surface deformation, and extensive filament formation (Fig. 2). Filamentation was observed in both honey- and antibiotic-treated cells but was substantially more pronounced at the bactericidal concentrations than at inhibitory concentrations. However, cell lysis was not observed in all treated samples.

To the best of our knowledge, this is the first study that showed filamentation of *C. acnes* upon treatments. However, there are other studies revealing filamentation of bacilli bacteria, such as *Escherichia coli* (*E. coli*) and *Bacillus subtilis* (*B. subtilis*) [15, 24]. The presence of filaments could be explained by *C. acnes* classification under the Actinobacteria phylum, known for their slow-growing nature and branching filamentous morphology [25, 26]. Among the six phylotypes (subtypes IA1, IA2, IB, IC, II and III) of *C. acnes*, phylotype III displays the characteristics of long and slender filaments [27]. In contrast, the non-filamentous coryneform (irregular rod-shaped) morphology was observed in *C. acnes* phylotypes I and II. However, the *C. acnes* strain in this study belongs to the subspecies (subsp.) acnes and phylotype IB that were reported to have no filaments, making the filamentation phenomenon remain unexplored [28, 29].

In FESEM, the absence of cell lysis can indicate the integrity of the damaged bacterial cell wall with the preserved cell membrane. The disruption of bacterial cell wall integrity distorts the curvy-rod shape of *C. acnes* and shifts to filamentous forms. As illustrated in Fig. 2 (B), (C), (D), and (E), the bacterial cells were shrunken and crumpled, deforming the bacterial rod shape and dispersing the filaments out from within the cells. This finding was similar to a study in which *H. itama* honey treatment resulted in cell shrinkage in the bacteria *B. subtilis*, *E. coli* and *P. aeruginosa*, where they were found to have reduced cell length while retaining their rod shapes [19]. The extent of bacterial cell deformation and degree of filamentation intensified with increasing honey concentrations from 20% to 40%, correlating with MIC and MBC treatments. At MIC treatment, the shorter filaments are postulated due to the preserved activity of penicillin-binding protein (PBP) 2, a protein involved in bacterial cell shape maintenance, in which some bacteria maintain their rod shapes instead of forming filaments [30].

Filamentation was stipulated to increase the fitness of microbes in response to stressors by averting cell rupture [31]. The possible reversion of bacterial cell shape and daughter cells’ maintained cell division capacity when the stressors are removed were demonstrated, suggesting the survival strategy of filamentation for over-stressed and dying bacteria [32]. For *E. coli* and *B. subtilis*, filamentation occurs as the bacterial cell division is halted to allow the continuation of lateral growth, leading to cell elongation without daughter cell separation [19]. From the molecular perspective, interrupting the SOS repair mechanism during DNA synthesis further halts the septum formation, leading to non-septated filamentation [19]. The bacterial cell deformation and filamentation appeared more severe in the honey-treated samples compared to the PCs (Fig. 2). This may be attributed to the MEM (PCs) being beta-lactams that mainly target the bacterial cell wall [33]. However, honey could target both the bacterial cell wall and DNA synthesis, further distorting the *C. acnes* bacterial cell shape into filamentous forms. As evidence, bacterial DNA damage was shown against *E. coli* upon Manuka and Yemeni Sidr honey treatment [34].

Honey’s high osmotic pressure, sugar concentration and acidity are some plausible causes of the shrinkage and filamentation of *C. acnes*. Generally, honey is a highly concentrated sugar solution consisting mainly of 17% water and 82.5% sugars, creating a hypertonic environment and halting bacterial proliferation [35]. The pH of honey in this study was in the range of 3.34 to 3.65, consistent with the findings by Zainin and Hassan (2021), who reported that the pH of Kelulut honey was as low as 3.24 to 3.71 [36]. Hence, the high acidity of the honey may further deteriorate the growing environment of *C. acnes*, which was reported to have optimal growth pH between 6 and 7, contributing to their shape deformation and filamentation [37].

### Liquid-Chromatography Mass Spectrometry (LCMS)

LCMS analysis identified phytochemical constituents in Kelulut honeydew honey based on SM-QTOF guidelines. Some of these phytochemicals, including purine, pteridine, theobromine, nebularine, and nigerose (sakebiose), were previously reported to exhibit antimicrobial activities. As listed in Table 2, both purine and pteridine derivatives showed antimicrobial activities against *S. aureus*, *E. coli* and *C. albicans* [38, 39]. Theobromine and nigerose exhibited potential antimicrobial activities against oral bacteria, *Streptococcus mutans* and *Streptococcus sanguinis*, respectively [40]. Whereas nebularine exhibited antimicrobial activities against various fungi and bacteria, respectively [41].This suggests that the antimicrobial efficacy of Kelulut honey arises from the synergistic interactions among multiple bioactive compounds rather than from a single dominant compound.

Collectively, the physicochemical properties and bioactive constituents of Kelulut honey underpin its selective yet potent antimicrobial activity, particularly against *C. acnes*.

**Table 2:** Known bioactive compounds of Kelulut dew honey with their known and uncharacterized (N/A) antimicrobial properties

Polarity	Compounds	Formula	Mass	Classification		Bioactivities	References
Negative	2-Hydroxy-2,4-pentadienoate	C_5_H_6_O_3_	114.0317	Fatty acid	N/A		
	Purine	C_5_H_4_N_4_	120.0436	Nucleoside	Novel series of purine benzimidazole hybrids showed antimicrobial activities against *S. aureus*, *Enterococcus faecalis*, *E. coli*, *Pseudomonas aeruginosa*, *Acinetobacter baumannii*, *C. albicans*, *Candida tropicalis*, *Aspergillus fumigatus*, *Candida parapsilosis*.		[38]
	Pteridine	C_6_H_4_N_4_	132.0434	Heterocyclic compound	Pteridine derivatives showed antimicrobial activities against *S. aureus*, *B. subtilis*, *E*. *coli*, *C. albicans*.		[39]
	Theobromine	C_7_H_8_N_4_O_2_	180.065	Alkaloids	Antimicrobial effects against *Streptococcus mutans*, *Lactobacillus acidophilus*, *Enterococcus faecalis* and *C. albicans*.		[40, 42]
	Nebularine	C_10_H_12_N_4_O_4_	252.0856	Nucleoside	Antifungal activities against *Magnaporthe grisea* and *Trichophyton mentagrophyte*.		[41]
	Asn-Asn-OH	C_13_H_14_N_4_O_8_	354.0815	Dipeptide	N/A		
	Fructoselysine 6-phosphate	C_12_H_25_N_2_O_10_P	388.124	Hexose phosphates	N/A		
	(3R,5 S,6E)-rel-7-[3-(4-fluorophenyl)−1 H-indol-2-yl]−3,5-dihydroxy-6-Heptenoic acid	C_21_H_20_FNO_4_	369.1375	Statin-like indole derivatives	N/A		
	Glucosylgalactosyl hydroxylysine	C_18_H_34_N_2_O_13_	486.2055	Glycosylated amino acid	N/A		
Positive	3-Hydroxy-3-methyl-glutaric acid	C_6_H_10_O_5_	162.0527	Dicarboxylic acid	No antimicrobial activities were reported but it promoted lipid and protein oxidative damage.		[43]
	L-Galactose	C_6_H_12_O_6_	180.0632	Carbohydrates	N/A		
	Starch acetate	C_12_H_18_O_9_	306.0949	Carbohydrate derivative	N/A		
	Nigerose (Sakebiose)	C_12_H_22_O_11_	342.116	Carbohydrates	Potential antimicrobial activities against *Streptococcus sanguinis*.		[40]
	Panose	C_18_H_32_O_16_	504.1686	Carbohydrates	N/A		

N/A, Non-available

## Conclusion

To conclude, Kelulut honeydew stingless bee honey holds the potential to be an effective base for natural acne treatment products. Notably, among the microbes, *C. acnes* exhibited the greatest susceptibility to honey treatment with the lowest honey MIC and MBC of 20% and 40% (v/v), respectively, underpinning the ability of honey to alleviate acne by reducing the overcolonization of *C. acnes*. In contrast, the other microbes (*S. aureus*, *S. epidermidis*, MRSA and *C. albicans*) were only inhibited but not eradicated by honey. The time-kill assays further validate honey’s time- and concentration-dependent antimicrobial effects against *C. acnes*. Intriguingly, *C. acnes* bacterial cell deformation and filamentation were observed under FESEM following exposure to honey. These findings lay the foundation for future research to delve deeper into the identity of *C. acnes* and its filamentation phenomenon. The synergistic effects of the honey’s bioactive ingredients may contribute to its notable antimicrobial efficacy. However, further studies are crucial to isolate and characterize the specific honey’s active ingredient(s) responsible for its antimicrobial activity. Future research could delve into the formulation and dosage optimization of honey as a topical anti-acne agent, alongside comprehensive evaluation of honey’s long-term safety and stability to develop a safe, natural, non-antibiotic-resistant alternative to conventional acne treatments.

## Data Availability

All data generated or analyzed during this study are included in this article.
